# “You Have Reached Your Destination”: A Single Trial EEG Classification Study

**DOI:** 10.3389/fnins.2020.00066

**Published:** 2020-02-11

**Authors:** Christopher Wirth, Jake Toth, Mahnaz Arvaneh

**Affiliations:** Automatic Control and Systems Engineering Department, University of Sheffield, Sheffield, United Kingdom

**Keywords:** EEG, classification, BCI, human machine interaction, neurophysiology, P300, navigation, target recognition

## Abstract

Studies have established that it is possible to differentiate between the brain's responses to observing correct and incorrect movements in navigation tasks. Furthermore, these classifications can be used as feedback for a learning-based BCI, to allow real or virtual robots to find quasi-optimal routes to a target. However, when navigating it is important not only to know we are moving in the right direction toward a target, but also to know when we have reached it. We asked participants to observe a virtual robot performing a 1-dimensional navigation task. We recorded EEG and then performed neurophysiological analysis on the responses to two classes of correct movements: those that moved closer to the target but did not reach it, and those that did reach the target. Further, we used a stepwise linear classifier on time-domain features to differentiate the classes on a single-trial basis. A second data set was also used to further test this single-trial classification. We found that the amplitude of the P300 was significantly greater in cases where the movement reached the target. Interestingly, we were able to classify the EEG signals evoked when observing the two classes of correct movements against each other with mean overall accuracy of 66.5 and 68.0% for the two data sets, with greater than chance levels of accuracy achieved for all participants. As a proof of concept, we have shown that it is possible to classify the EEG responses in observing these different correct movements against each other using single-trial EEG. This could be used as part of a learning-based BCI and opens a new door toward a more autonomous BCI navigation system.

## 1. Introduction

Studies concerning robotic movement and navigation tasks have previously used electroencephalography (EEG) to investigate the brain's responses to observing correct and erroneous movements. These studies have shown that it is possible to classify the responses to correct movements against erroneous ones on a single-trial basis (Chavarriaga et al., 2014; Iturrate et al., 2015; Zander et al., 2016; Kim et al., 2017). Furthermore, a few recent studies have demonstrated the feasibility of using such correct-vs-error classification as feedback for reinforcement-learning-based Brain-Computer Interfaces (BCI) (Iturrate et al., 2015; Zander et al., 2016; Kim et al., 2017). Additionally, some studies have shown that different erroneous conditions can be classified against each other (Iturrate et al., 2010; Spüler and Niethammer, 2015; Wirth et al., 2019). These interesting advances have created the possibility of systems in which machines can control the low-level action decisions in order to navigate semi-autonomously toward a target, with feedback provided via implicit communication with a user through brain signals spontaneously generated while observing the task (Iturrate et al., 2015; Zander et al., 2016).

However, none of these previous studies have investigated whether it is possible to classify EEG responses to different types of correct actions against each other. In most navigation tasks, it is crucial not only to know that you are moving in the correct direction, but also to recognize when you have reached your destination. As such, it is highly important to consider whether there are significant neurophysiological differences between the brain's responses to observing different correct movements: those that get closer to a target, compared to those that actually reach it.

To address this question, we evaluated data from a virtual robotic navigation task. Participants were asked to observe a virtual robot, represented by a cursor, navigating in a 1-dimensional space and attempting to reach a target. We then investigated the EEG responses to movements that reached the target (hereafter referred to as the “TR condition,” short for “target reached”), in contrast to the responses to movements toward the target, but not reaching it (hereafter referred to as the “TT condition,” short for “toward target”).

To explore neurophysiologicial distinctions between the TT condition and the TR condition, we used time domain features to compare the latency and amplitude of key features of the event related potentials (ERPs). We also examined the spatial distribution of EEG responses to each condition, using topographical maps.

Our main focus was on the P300: a positive peak in an ERP at ~300 ms (Smith et al., 1970; Picton, 1992), known to be elicited in the brain when a subject recognizes a target stimulus in a sequence containing both target and non-target stimuli (Polich et al., 1991, 1996; Picton, 1992). The P300 has been successfully utilized in BCI, notably in spelling devices (Farwell and Donchin, 1988; Sellers and Donchin, 2006; Krusienski et al., 2008; Gugera et al., 2009; Fazel-Rezai et al., 2012). In these cases, the “target” stimulus is the specific character the user wishes to type. Each potentially desired character is typically highlighted a number of times, with each time being referred to as a “subtrial.” These subtrials are then averaged to increase the robustness of classification (Farwell and Donchin, 1988; Sellers and Donchin, 2006; Lotte et al., 2007; Krusienski et al., 2008; Gugera et al., 2009; Fazel-Rezai et al., 2012). Similar systems have also been developed for the control of robots (Lüth et al., 2007; Bell et al., 2008; Johnson et al., 2010; Bhattacharyya et al., 2014), cursors (Polikoff et al., 1995; Li et al., 2010; Kanoh et al., 2011), and wheelchairs (Rebsamen et al., 2006; Iturrate et al., 2009).

Unlike these previous studies utilizing the P300 for robotic control, and similar applications, in our study each stimulus (i.e., each movement) was only presented once, and so our classification phase required single-trial classification. Single-trial P300 classification is challenging, due in part to the low signal-to-noise ratio of EEG data (Jansen et al., 2004; Lotte et al., 2007), hence many systems presenting a number of subtrials. One study investigated the effects of different numbers of subtrials, and, while high accuracy was achieved with many subtrials, classification accuracy of <50% was reported based on a single subtrial, and 3 subtrials were required to achieve over 60% accuracy (Lenhardt et al., 2008). More recently, studies focusing on single-trial P300 classification have shown success, with some reporting accuracies over 80% (Finke et al., 2009; Korczowski et al., 2015; Lin et al., 2017). These studies were classifying the presence of a P300 against its absence. Our goal was to differentiate the P300s elicited in response to two slightly different desired actions. This presents an extra challenge, as we can expect the signals of the conditions to be more similar to each other.

In one previous study, one version of a task presented 80% standard stimuli and 20% target stimuli with all targets being identical to each other, while another version presented 80% standard stimuli and 20% target stimuli, with a pool of 25 different target stimuli; the latter case was found to elicit a broader P300 (Breton, 1988). While the responses to the different target stimuli were not compared to one another, this finding suggests that the P300 is affected by how often a specific stimulus appears in a task. Indeed, other literature has reported that P300 amplitude increases for larger target-to-target intervals (Gonsalvez and Polich, 2002). As well as this, the P300 has been shown to be associated with positive outcomes (Hajcak et al., 2005), and its amplitude has been shown to be affected by reward magnitude (Yeung and Sanfey, 2004; Sato et al., 2005; Wu and Zhou, 2009).

In this study, the desired stimulus is either a movement toward the target or, in cases when the virtual robot is adjacent to the target location, a movement that reaches the target. We hoped to identify and exploit differences between responses to these stimuli, arising from both the experimental differences (i.e., reaching the target occurs less frequently than other correct moves) and the participants' cognitive response to the two conditions (i.e., reaching the target may be considered more important than other correct moves). We then aimed to use the identified neurophysiological differences in order to classify the EEG responses to the two conditions against each other on a single-trial basis.

In order to classify responses to the conditions against each other, we implemented a stepwise linear discriminant analysis strategy, using time domain features from six electrode sites to generate subject-specific classification models. A second publicly available data set (Chavarriaga and Millán, 2010), gathered from participants observing a similar 1-dimensional navigation paradigm, was used to further validate the efficacy of the classification strategy. We tested our approach using data from 10 healthy young adults from the first task, and a further five healthy young adults from the second task.

## 2. Methods

This study uses data from two tasks. Neurophysiological analysis and single-trial classification were performed on data from Task 1. These data were recorded at the University of Sheffield, UK. Data from a Task 2 were used in order to further validate the single-trial classification section of the study. This was an open access data set, obtained under a Creative Commons Attribution—Non Commercial—No Derivatives 4.0 International license, based on a study by Chavarriaga and Millán (2010).

### 2.1. Participants

Ten healthy adults (4 female, 6 male, mean age 27.30 ± 8.31) were recruited to participate in Task 1. All of these participants were included in all aspects of the study. All participants had normal or corrected-to-normal vision. They reported no history of psychiatric illness, head injury, or photosensitive epilepsy. Written informed consent was provided by all participants before testing began. All procedures were in accordance with the Declaration of Helsinki, and were approved by the University of Sheffield Ethics Committee in the Automatic Control and Systems Engineering Department.

Six healthy adults (1 female, 5 male, mean age 27.83 ± 2.23) performed Task 2. 1 participant was excluded from this study as too few trials were available after artifact rejection.

### 2.2. Experimental Setup

#### 2.2.1. EEG Setup

For Task 1, eight channels of EEG were recorded at 500 Hz using an Enobio 8 headset. The electrode sites recorded were Fz, Cz, Pz, Oz, C3, C4, P07, and PO8. A further reference electrode was placed on the earlobe.

For Task 2, 64 channels of EEG were recorded at 512 Hz using a BioSemi ActiveTwo system, and were referenced to the common average. Electrodes were placed using the 10–20 system.

#### 2.2.2. Task 1

**Figure 1:**
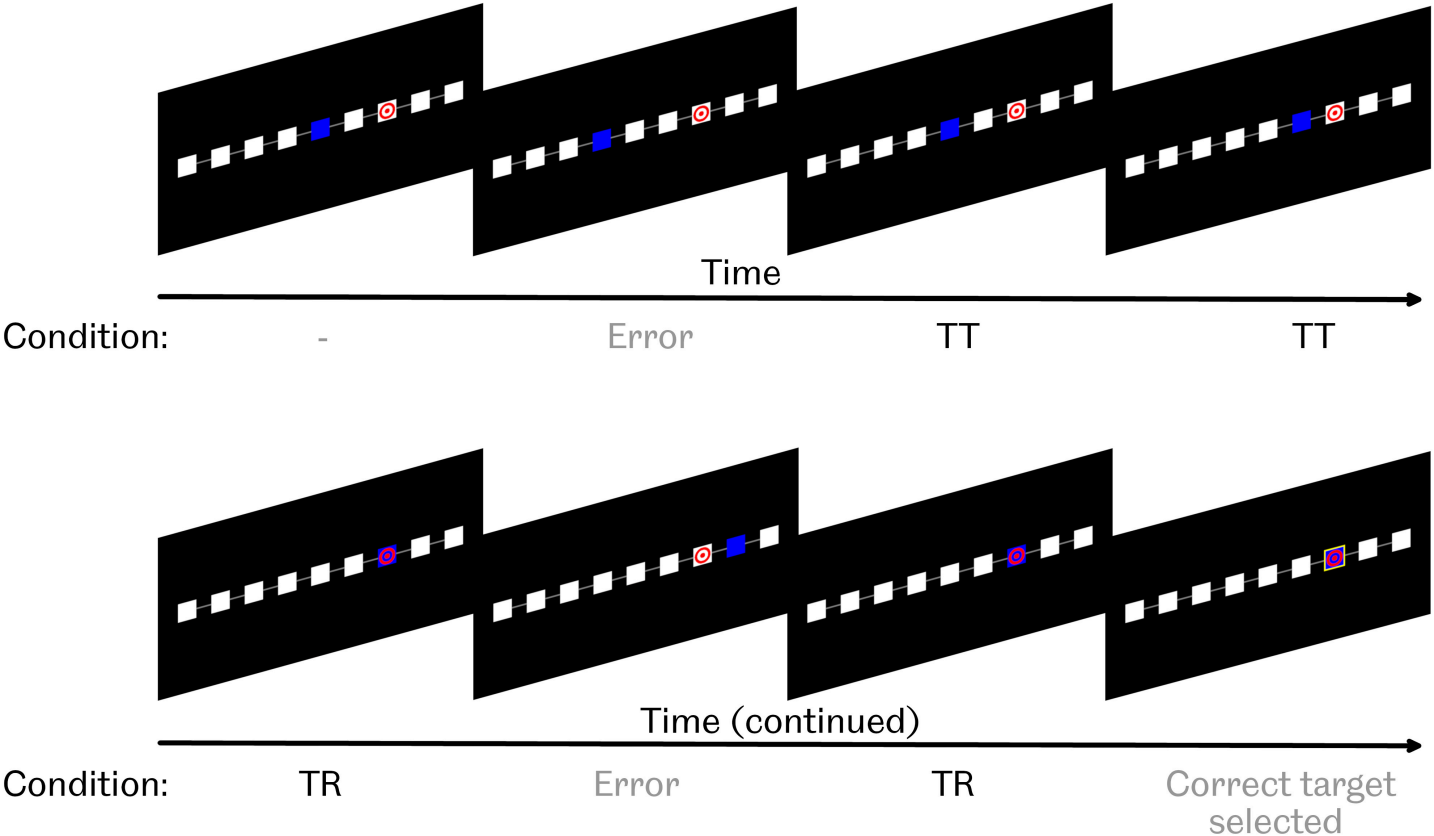
Task 1 paradigm. Participants were asked to observe as a blue cursor attempted to navigate toward, and select, a marked target square. If the cursor was on the target, possible actions were either to select it by drawing a yellow box around the square, or take 1 step away from the target. If the cursor was not on the target, possible actions were either to move 1 step toward the target, move 1 step further away from the target, or erroneously select the current square as the target by drawing a yellow box around it. “TT” condition refers to “toward target,” i.e., movements toward, but not reaching, the target. “TR” condition refers to “target reached,” i.e., movements that did reach the target.

In Task 1, participants were seated in front of a screen and asked to observe a computer controlled cursor. Participants were presented with nine squares, arranged in a horizontal line, on a black background, as seen in Figure 1. The cursor's current square was colored blue. The target square was identified by a red bullseye symbol on a white background. All other squares were plain white.

At the beginning of each run, the cursor appeared 2 or 3 squares away from the target location, either to the left or the right. Every 2 s, either the cursor would move to an adjacent square, or a yellow box would be drawn around the cursor's current position in order to identify that the computer believed that it had reached the target. Such target identification could occur correctly or erroneously. Actions occurred with preset probabilities, which depended on whether or not the cursor was on the target. These probabilities are shown in Table 1.

**Table 1:** Action probabilities for Tasks 1 and 2.

**Task**	**Cursor location**	**Action**	**Probability**
		Move toward target	0.7
	Not on target	Move further from target	0.2
Task 1		Identify location as target	0.1
	On target	Identify location as target	0.67
		Step off target	0.33
Task 2	Not on target	Move toward target	0.8
		Move further from target	0.2

*Note that each run in Task 2 ended once the cursor reached the target. As such there were no moves from an on-target position in Task 2. In both tasks, both TT and TR conditions occurred as a result of “move toward target” actions. If these actions occurred when the cursor was adjacent to the target, the result would be reaching the target (i.e., TR condition). If the cursor was not adjacent to the target prior to the action, the result would be moving closer to the target, but not reaching it (i.e., TT condition)*.

After the target was identified, either correctly or erroneously, the run finished and the screen was cleared. After 5 s, the next run began. A beep sounded 1 s before the start of each run. Participants were asked to refrain from movement and blinking during each run, but told that they could move and blink freely between runs, while the screen was blank. This process repeated until the end of the block, with each block lasting ~4 min.

Each participant performed a single session of observations. Participants were asked to observe blocks, with breaks of as long as they wished between blocks, until they reported their concentration levels beginning to decrease. Most participants observed six blocks of trials. However, two participants observed only 2 blocks. On average, Task 1 participants observed a total of 149.2 ± 40.0 (mean ± standard deviation) TT condition trials, and 82.3 ± 20.0 TR condition trials.

#### 2.2.3. Task 2

In Task 2, participants were similarly asked to observe the 1-dimensional movement of a computer-controlled cursor. Twenty locations were arranged in a horizontal line across a screen. The cursor was displayed as a green square. The target was displayed as a blue square when it appeared to the left of the cursor, or a red square when it appeared to the right of the cursor.

At the beginning of a run, the target was drawn no more than three positions away from the cursor. Every 2 s, the cursor would move either toward or away from the target with preset probabilities, shown in Table 1. Unlike Task 1, no target identification was required by the computer. Instead, each run ended when the cursor reached the target. After this, the cursor stayed in its existing location, and a new target was drawn, again no more than three positions away from the cursor. This process repeated until the end of the block, with each block lasting 3 min.

Participants each performed two sessions of observations. Each session consisted of 10 blocks. The number of days between sessions varied between participants, from a minimum of 50 days to a maximum of more than 600 days. On average, Task 2 participants observed a total of 620.2 ± 10.6 TT condition trials, and 277.7 ± 14.1 TR condition trials.

### 2.3. Neurophysiological Analysis

Data from Task 1 were used for neurophysiological analysis. As we did not have control over the experimental paradigm for Task 2, and so did not have a precisely detailed picture of how the stimuli were presented, we opted not to perform neurophysiological analysis on Task 2 data, instead using these only to further validate the classification phase of this study.

Raw data from Task 1 were resampled to 64 Hz, and then band-pass filtered from 1 to 10 Hz, using a zero-phase Butterworth filter. TT and TR Trials were extracted from a time window of 0 to 1,000 ms, relative to the movement of the cursor. All extracted trials were baseline corrected relative to a period of 200 ms immediately before the movement of the cursor. Artifact rejection was performed by discarding any trials in which the range between the highest and lowest amplitudes, in any channel, was >100μV.

Grand average time domain event related potential (ERP) data were plotted using the extracted trials, showing the mean voltage ± 1 standard error, comparing responses to the TT condition with those to the TR condition.

Peak analysis was performed in order to identify the latencies at which the P300 occurred in the ERP data. Visual inspection of time domain ERP and topographical plots indicated that the highest P300 amplitude in this study occurred at electrode site Cz, and that there was a difference in P300 amplitudes in response to the two conditions at this site. As such, Cz was chosen as the most suitable channel for peak analysis. This peak analysis was carried out on the grand average ERP for responses to each condition. Subsequently, the P300 was identified as the highest positive peak, occurring between 200 and 500 ms. This time window was selected based on a visual inspection of the grand average time-domain data. To check for statistically significant differences in peak latencies, the same analysis was carried out to find the P300 peak in the average responses of each individual participant, for both conditions. According to one-sample Kolmogorov-Smirnov tests, we could not assume the data to be normally distributed. Therefore, a Wilcoxon signed-rank test was performed to compare the peak latencies identified for the two conditions.

To check whether there was a statistically significant difference in peak amplitude between responses to the two conditions, the mean amplitude was calculated in the responses the average responses of each individual participant, in a time window from 200 to 500 ms in order to encapsulate the full breadth of the P300. According to one-sample Kolmogorov-Smirnov tests, we could not assume the data to be normally distributed. Therefore, a Wilcoxon signed-rank test was performed to compare the amplitudes identified for the two conditions.

Topographical maps were then plotted for responses to each condition, using a 50 ms window surrounding the P300 latency (from peak −25 ms to peak +25 ms) as identified in the pooled data from all trials of both conditions combined. All topographical maps used the same scale, from the minimum value to the maximum values across all grand averages.

### 2.4. Single-Trial Classification

Single-trial classification was performed on data from both tasks. The same classification protocol was followed for both data sets, and is described in this section.

#### 2.4.1. Pre-processing and Feature Extraction

Data from six electrode sites were used for single-trial classification: Fz, Cz, Pz, Oz, PO7, and PO8. These channels were selected based on visual inspection of grand average time domain ERPs, and considering prior knowledge related to these sites. The P300 has shown to peak in midline electrodes (Polich et al., 1997), and posterior sites, such as PO7 and PO8 are associated with visual processing (Deutsch et al., 1988; Wolber and Wascher, 2005; Schneider et al., 2012). As with the neurophysiological analysis, data were resampled at 64 Hz, trials were baseline corrected to a period of 200 ms immediately before presentation of the stimulus, and artifact rejection was performed to remove any trials with a range of >100μV between the highest and lowest amplitude in any channel. For the classification phase, data were band-pass filtered between 1 and 32 Hz. This band was selected after visual inspection of event-related spectral perturbation (ERSP) data which showed that, while most activity occurred at low frequencies, some potentially useful activity was also present in higher frequencies (see Supplementary Figure 1). Trials were extracted from 200 to 700 ms relative to the movement of the cursor. This window was selected based on visual inspection of grand average time domain ERPs. Selecting this window results in 33 samples per channel. Thus, in total, each trial was represented by 198 (6 × 33) features.

Previous literature has suggested that a minimum of 20 trials are required to provide stability in the P300 (Cohen and Polich, 1997). As such, we implemented a minimum cut-off of 20 artifact-free trials per class, in order to ensure we had enough data to produce a reliable training set. One participant was excluded from the single-trial classification phase of this study due to this cutoff.

#### 2.4.2. Classification With Stepwise Linear Discriminant Analysis

In order to classify the data based on the most relevant subset of features, stepwise linear discriminant analysis was chosen as our classification approach, as previous literature has shown this strategy to be effective at both feature selection and classification of both P300 (Donchin et al., 2000; Krusienski et al., 2006, 2008; Sellers and Donchin, 2006; Lotte et al., 2018) and motion-onset visual evoked potential (mVEP) EEG data (Guo et al., 2008). An individual classification model was generated for each participant, using only the data from that individual participant's responses to the task. Firstly, for a given participant, an initial subset of features was selected. The amplitudes of the training trials for each condition were compared in each feature (i.e., each combination of channel and time point) using an unequal variances *t*-test. Features whose *p*-value was < 0.05 were included in the initial feature set. The stepwise procedure was then performed to select which features would be included in the final model. At each step, a regression analysis was performed on models with and without each feature, producing an F-statistic with a *p*-value for each feature. If the *p*-value of any feature was < 0.05, the feature with the smallest *p*-value would be added. Otherwise, if the *p*-value of any features already in the model had risen to >0.10 at the current step, the feature with the largest *p*-value would be removed from the model. This process continued until no feature's *p*-value reached the thresholds for being added to, or removed from, the model. If no features were added to the model at all, a single feature with the smallest *p*-value would be selected. Training and test trials were then reduced to the selected features.

The training set for the condition with the fewest training trials was oversampled in order to ensure that training occurred with an equal number of trials per condition. A linear classification model was then trained and tested. All classifiers were trained and tested using leave-one-out cross validation. To test statistical significance of the classification, a right-tailed Fisher's exact test was performed on the confusion matrix of each participant's results. In order to test whether the classification was significant at a group level, individual *p*-values were combined into a group *p*-value using Fisher's method (Loughin, 2004; Heard and Rubin-Delanchy, 2018).

## 3. Results

### 3.1. Neurophysiological Distinctions

**Figure 2:**
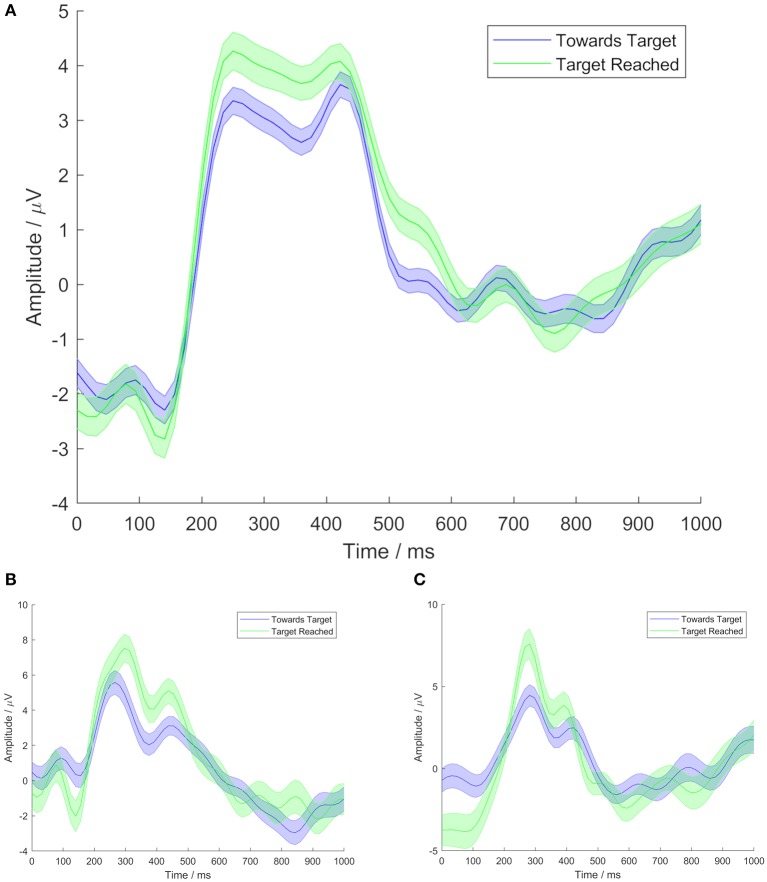
Time domain ERPs at electrode site Cz, from Task 1. Time shown is relative to movement of the cursor. Central lines represent mean signals. Shaded areas cover 1 standard error. Blue lines show TT condition data. Green lines show TR condition data. **(A)** Shows grand average data from all Task 1 participants, **(B)** shows data from participant 1, and **(C)** shows data from participant 10.

In the responses to both conditions, grand average time domain ERPs showed a broad P300 peak, as can be seen in Figure 2A. Figures 2B,C show examples of time domain ERPs from individual participants (1 and 10, respectively). In both conditions, the shape of the broad P300 featured a peak shortly prior to 300 ms, followed by a slight drop in amplitude, and then a secondary peak, shortly after 400 ms. In responses to the TR condition, the earlier peak was found to have the highest amplitude, at a latency of 265 ms. The secondary peak marked the highest amplitude in grand average responses to the TT condition, with a latency of 420 ms. However, the Wilcoxon signed-rank test did not find a significant difference between the P300 peak latencies of responses to the two conditions (*p* = 0.81).

A distinction was seen between the P300 amplitudes of responses to the two conditions. The TR condition was observed to elicit a P300 with a greater amplitude than that generated in response to the TT condition. The Wilcoxon signed-rank test comparing the amplitudes of the two conditions, based on a time window from 200 to 500 ms in order to encapsulate the breadth of the P300, found this difference in amplitude to be statistically significant (*p* = 0.004).

Grand average time domain data for all eight electrode sites recorded for Task 1 are shown in Supplementary Figure 2.

**Figure 3:**
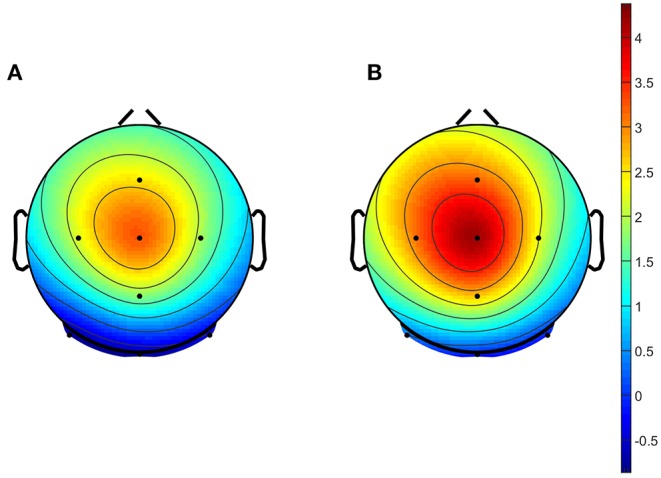
Grand average topographical maps of Task 1 data. Maps were plotted based on a 50 ms window surrounding the peaks identified as P300 from grand average data across all participants, and both conditions. Plots shown represent **(A)** responses to the TT condition, and **(B)** responses to the TR condition.

Topographical maps plotted at the P300 peak latency showed the main activation to occur in the central midline, in response to both conditions, as can be seen in Figure 3.

We observed some features in the ERP responses to both conditions which may be related to motion-onset visual evoked potentials (mVEP). Such mVEPs occur when users percieve the beginning of movement of an object or symbol on a screen (Kuba et al., 2007; Guo et al., 2008; Marshall et al., 2013; Beveridge et al., 2019). Three main peaks have been identified in mVEP: a positive peak (P1), followed by a negative deflection (N2), then another positive peak with a latency of 240–500 ms (Kuba et al., 2007; Guo et al., 2008; Marshall et al., 2013; Beveridge et al., 2019), which has been described as a P2 (Kuba et al., 2007; Guo et al., 2008; Marshall et al., 2013) or P300 (Beveridge et al., 2019). The movements considered in this study were instantaneous steps from one location to the next. However, along with the P300, small P1 and N2 peaks were visible, with latencies of 78 and 125 ms, respectively, relative to the movement of the cursor. These peaks did not appear to differ between responses to the two conditions.

### 3.2. Classification

#### 3.2.1. Classification of Task 1

The classification accuracies of each individual participant of Task 1 are shown in Table 2. The mean overall accuracy for all Task 1 participants was 66.5%. The mean accuracy for the TT condition was 68.8%, and the mean accuracy for the TR condition was 62.4%. Statistically significant separation of the conditions (*p* < 0.05) was found for all Task 1 participants. At a group level, the classification results for Task 1 were found to be statistically significant (*p* = 2.8 × 10^−54^).

**Table 2:** Single-trial classification results of Task 1 data.

**Subject**	**# TT**	**# TR**	**Mean # Features**	**TT**	**TR**	**Overall**	***p*-value**
	**trials**	**trials**	**selected**	**accuracy (%)**	**accuracy (%)**	**accuracy (%)**	
1	162	86	35.0	64.8	55.8	61.7	1.4 × 10^−3^
2	73	40	44.8	68.5	60.0	65.5	3.1 × 10^−3^
3	157	93	10.3	60.5	51.6	57.2	4.2 × 10^−2^
4	163	89	50.9	76.1	70.8	74.2	4.1 × 10^−13^
5	63	39	30.4	65.1	53.8	60.8	4.7 × 10^−2^
6	155	88	6.3	67.7	63.6	66.3	1.9 × 10^−6^
7	154	85	16.6	59.7	52.9	57.3	4.0 × 10^−2^
8	156	81	15.9	67.3	61.7	65.4	1.7 × 10^−5^
9	145	76	40.0	73.1	72.4	72.9	7.5 × 10^−11^
10	169	89	37.7	85.2	80.9	83.7	5.0 × 10^−26^
							**All** < **0.05**,
**Mean**	**139.7**	**76.6**	**28.8**	**68.8**	**62.4**	**66.5**	**group** ***p*****-value:**
							*p* = 2.8 × 10^−54^

*Overall accuracy calculated as the percentage of trials, of either class, correctly classified. Number of features selected calculated as the mean of all iterations of leave-one-out cross-validation*.

#### 3.2.2. Classification of Task 2

The classification accuracies of each individual participant of Task 2 are shown in Table 3. The mean overall accuracy for all Task 2 participants was 68.0%. The mean accuracy for the TT condition was 70.5%, and the mean accuracy for the TR condition was 61.0%. As with Task 1, statistically significant separation of the conditions (*p* < 0.05) was found for all Task 2 participants. At a group level, the classification results for Task 2 were found to be statistically significant (*p* = 9.6 × 10^−62^).

**Table 3:** Single-trial classification results of Task 2 data.

**Subject**	**# TT**	**# TR**	**Mean # Features**	**TT**	**TR**	**Overall**	***p*-value**
	**trials**	**trials**	**selected**	**accuracy (%)**	**accuracy (%)**	**accuracy (%)**	
1	448	105	44.3	75.0	60.0	72.2	1.9 × 10^−11^
2	585	180	89.2	74.2	66.7	72.4	7.3 × 10^−23^
3	259	128	64.7	67.6	60.9	65.4	8.4 × 10^−8^
4	201	93	31.5	61.7	51.6	58.5	2.2 × 10^−2^
5	603	250	71.8	74.0	66.0	71.6	1.4 × 10^−27^
							**All** < **0.05**,
**Mean**	**419.2**	**151.2**	**60.3**	**70.5**	**61.0**	**68.0**	**group** ***p*****-value:**
							*p* = 9.6 × 10^−62^

*Overall accuracy calculated as the percentage of trials, of either class, correctly classified. Number of features selected calculated as the mean of all iterations of leave-one-out cross-validation*.

## 4. Discussion and Conclusion

### 4.1. Neurophysiological Distinctions Between the Conditions

In this study, the key neurophysiological difference that we identified between the two conditions was in the amplitude of the P300. The amplitude of the P300 was found to be greater in response to the TR condition (i.e., movements that reached the target) than the TT condition (i.e., movements that were correct, but did not reach the target). This distinction was found to be statistically significant (*p* = 0.004).

As discussed in section 1, a number of studies have reported that P300 amplitude is affected by reward magnitude (Yeung and Sanfey, 2004; Sato et al., 2005; Wu and Zhou, 2009). It should be noted that, in this study, participants were not directly rewarded based on the virtual robot's performance. However, it is certainly feasible that they regarded moves that reached the target as being more important than moves that did not reach it, which could be considered analogous to the TR condition having a higher reward magnitude. Reports have been mixed regarding the effects of valence on the P300. Some studies have reported amplitude being affected by positive valence (Cano et al., 2009; Wu and Zhou, 2009), while others have reported valence either having no effect (Carretié et al., 1997; Yeung and Sanfey, 2004), or an effect only in the case of negative valence (Conroy and Polich, 2007). P300 amplitude has also been shown to be dependent on whether feedback was expected or unexpected (Hajcak et al., 2005), and on target-to-target interval, with amplitude increasing when targets appeared less frequently (Gonsalvez and Polich, 2002).

Taking into consideration previous findings on the P300, and the experimental setup of our task, there are a number of potential causes of this increase in amplitude for responses to the TR condition, compared to the TT condition. It may represent a cognitive response recognizing that a move that reaches the target is a more important step than other correct moves. Alternatively, while this study was designed as a navigation observation task, it could also conceptually be considered as an oddball paradigm. That is to say, the TR condition occurs less frequently than the TT condition. Therefore, it is possible that the increased P300 amplitude is due to the relative rarity of the TR condition. It is quite possible that the difference in amplitude may be the result of a combination of these factors.

We also briefly investigated frontal theta power, and asymmetry in alpha power, as these have been reported to vary with regard to valence (Reuderink et al., 2013). However, no significant differences in these markers were identified between the conditions. It is certainly feasible that participants would not have had a strong emotional reaction to reaching the target. In Task 1, the goal was not fully achieved until the target was not only reached but also identified. Furthermore, users knew they were not controlling the virtual robot, and were not rewarded if it performed well. It may be interesting to investigate whether these valence markers indicate different reactions in future on-line experiments, in which participants' responses affect the actions of the virtual robot.

### 4.2. Single-Trial Classification

Previous studies have successfully classified the brain's responses to correct movements against responses to erroneous movements in navigation tasks, such as the ones explored in this study. The original study for which the data of Task 2 were generated reported classification accuracy of 75.8 and 63.2% for the correct and erroneous movement classes, respectively (Chavarriaga and Millán, 2010). Another study reported correct vs. erroneous movement classification accuracy, in three similar navigation tasks, of 73.8, 72.5, and 74.3% (Iturrate et al., 2015). It is reasonable to expect that the classification of two different correct movements against each other would be more challenging than the classification of correct movements against erroneous ones; we would expect to see more pronounced differences in the brain's responses in the latter case.

In this study, classifying EEG responses to correct movements toward the target (but not reaching it) against responses to movements that reached the target, we achieved mean overall classification accuracy of 66.5 and 68.0% for the two tasks. Indeed, these were only slightly below the levels previously reported for erroneous vs. correct movements in similar tasks. Interestingly, overall accuracy reached a high of 83.7% in the best case. Crucially, statistically significant separation of the two conditions (*p* < 0.05) was achieved for all participants from both tasks, and highly significant separation of the classes was shown at the group level (*p* = 2.8 × 10^−54^ and *p* = 9.6 × 10^−62^ for the Task 1 and Task 2, respectively).

As a proof of concept, we have shown that it is possible to classify responses to these two classes of correct movement against each other using single-trial EEG. As discussed in section 2.4.2, we chose to apply stepwise linear discriminant analysis in this study, as it has previously been shown to be successful in classifying similar data types (Donchin et al., 2000; Krusienski et al., 2006, 2008; Sellers and Donchin, 2006; Guo et al., 2008; Lotte et al., 2018). However, it is possible that other methodologies, which could be explored in future, may be able to provide further increases in classification accuracy. In potential future systems, classifications of the human observer's EEG responses could be used to guide the movement of a real or virtual robot, with the user being explicitly rewarded for good performance of the robot. In such systems, adding information from more frontal electrodes may be able to provide an increase in classification accuracy, as the frontal cortex has been shown to code prediction and reward (Schultz et al., 1997; Schultz, 2001; McClure et al., 2004).

### 4.3. Implications for BCI

The P300 has a history of successful use in BCI, as discussed in section 1. In particular, there have been many studies, dating back over 30 years, regarding the use of P300 signals in BCI spelling devices (Farwell and Donchin, 1988; Sellers and Donchin, 2006; Krusienski et al., 2008; Gugera et al., 2009; Fazel-Rezai et al., 2012). These systems have often been able to improve the robustness and accuracy of their classifications by using paradigms that allowed each stimulus to be presented multiple times, and the responses to be averaged. P300-based BCIs have also been created for other applications, such as video games (Finke et al., 2009; Kaplan et al., 2013), virtual reality (Bayliss, 2003), and control of robots (Lüth et al., 2007; Bell et al., 2008; Johnson et al., 2010; Bhattacharyya et al., 2014), cursors (Polikoff et al., 1995; Li et al., 2010; Kanoh et al., 2011) and wheelchairs (Rebsamen et al., 2006; Iturrate et al., 2009). Furthermore, the P300 has been utilized alongside other modalities, such as motor imagery (Su et al., 2011) and steady-state visual evoked potentials (SSVEP) (Yin et al., 2013) to create hybrid BCIs (Pfurtscheller et al., 2010; Müller-Putz et al., 2011; Amiri et al., 2013). The navigation scenarios presented in this study provided a further challenge compared to many previous P300-related systems, as each stimulus (i.e., movement) was only presented once. This was an important aspect of the paradigm, as we wished to simulate the observation of real navigation, with a view to future applications in which classifications could be made solely based on users' responses to the actions they observe. In such real navigation, each action occurs only once. While accurate single-trial P300 classification is challenging due to the low signal-to-noise ratio of EEG (Jansen et al., 2004; Lotte et al., 2007), some recent studies have shown that it can be achieved. One study using a video game context reported mean offline classification accuracy of 85%, and online accuracy of 66% (Finke et al., 2009). Another study reported single trial P300 classification accuracy of 70% (Jansen et al., 2004). In other cases, the area under the receiver operating characteristic curve (AUC) was reported for various possible classifier parameters, rather than the classification accuracy for a specific trained and optimized model. An AUC of over 0.8 has been reported for many participants (Korczowski et al., 2015; Lin et al., 2017). In this study, rather than classifying a condition eliciting a P300 against a condition that did not elicit a P300, we were classifying two P300-generating conditions against each other. As such the fact that statistically significant separation of two different correct conditions was achieved for all participants is encouraging for the use of the P300 in single-trial BCI scenarios.

In recent years, there have been interesting advances in BCIs based on signals that are generated spontaneously in the brain, without the need of a conscious effort to generate them on the part of the user. These systems, making use of implicit communication, have been described in two groups, referred to as “reactive BCI,” in which a spontaneous response is triggered by a stimulus, and “passive BCI,” whereby arbitrary mental states are measured (Zander et al., 2010, 2014; Zander and Köthe, 2011). Some particularly interesting recent studies have been those exploring reactive BCI in robotic movement and navigation tasks. Classification of error-related potentials (ErrP) in order to differentiate correct movements from erroneous ones has been combined with reinforcement learning in order to allow machines to perform a desired action (Kim et al., 2017) or navigate toward a desired target (Chavarriaga and Millán, 2010; Iturrate et al., 2015; Zander et al., 2016). By obtaining more detailed information from spontaneously generated signals, we can provide these systems with more context, and allow them to learn more efficiently and act more appropriately. The ability to classify when a target has been reached specifically and separately from other correct movements, as has been demonstrated in this study, would be an important aspect of a navigation system, and thus could enhance the usability and effectiveness of navigation-based BCI.

### 4.4. Conclusion

In this study, we compared the ERPs generated in EEG data, in response to observing two types of correct movements by a virtual robot: those that moved the robot closer to the target without reaching it, and those in which the robot reached the target. We were able to show that both correct movement conditions elicited a P300, and we identified a significantly higher P300 amplitude in cases in which the target was reached.

Interestingly, we were able to classify the responses to these two types of correct actions against each other with mean overall accuracies of 66.5 and 68.0% for two tasks, achieving statistically significant separation of the conditions for all participants. This single-trial classification could be used as part of a learning-based BCI, and opens a new door toward a more autonomous BCI navigation system.

## Data Availability Statement

The raw data supporting the conclusions of this article will be made available by the authors, within 3 years, once further experiments have concluded, to any qualified researcher.

## Ethics Statement

The studies involving human participants were in accordance with the Declaration of Helsinki, and were reviewed and approved by University of Sheffield Ethics Committee in the Automatic Control and Systems Engineering Department. The participants provided their written informed consent to participate in this study.

## Author Contributions

MA conceived the study, supervised the work, and contributed to writing the paper. JT performed the data collection for Task 1. CW designed Task 1, performed the neurophysiological analysis and single-trial analysis, and wrote the paper.

### Conflict of Interest

The authors declare that the research was conducted in the absence of any commercial or financial relationships that could be construed as a potential conflict of interest.
