# Supplementary figures and images for: “You Have Reached Your Destination”: A Single Trial EEG Classification Study
Source: Front Neurosci. 2020 Feb 11;14:66. doi: 10.3389/fnins.2020.00066 (PMC7027274; doi:10.3389/fnins.2020.00066)

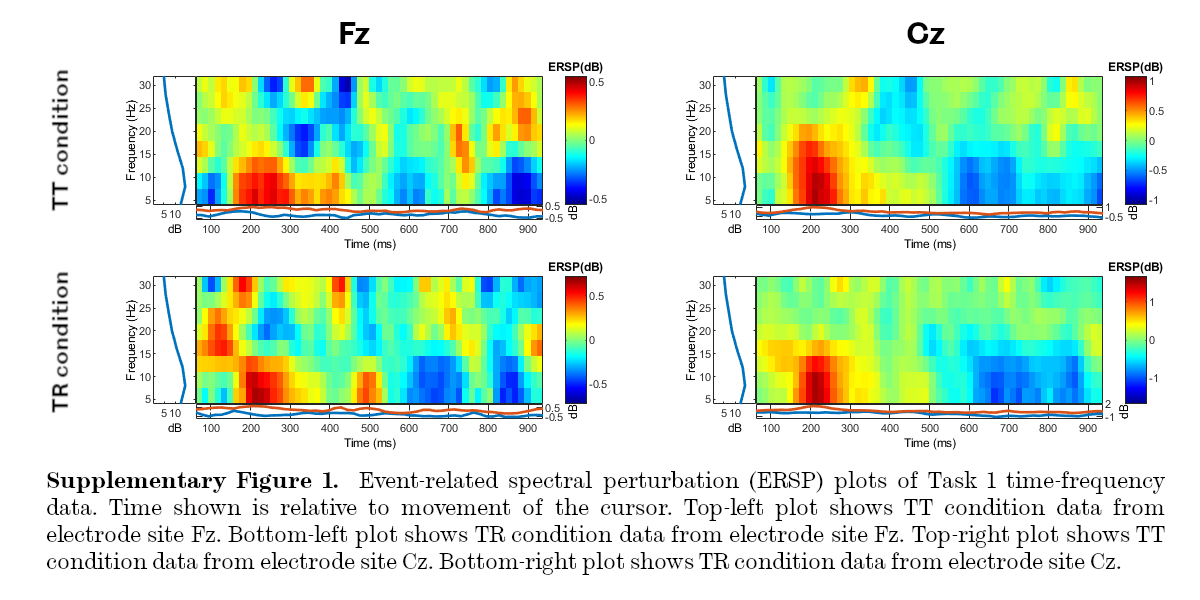

Supplement: Supplementary file 1 [file Image_1.JPEG]

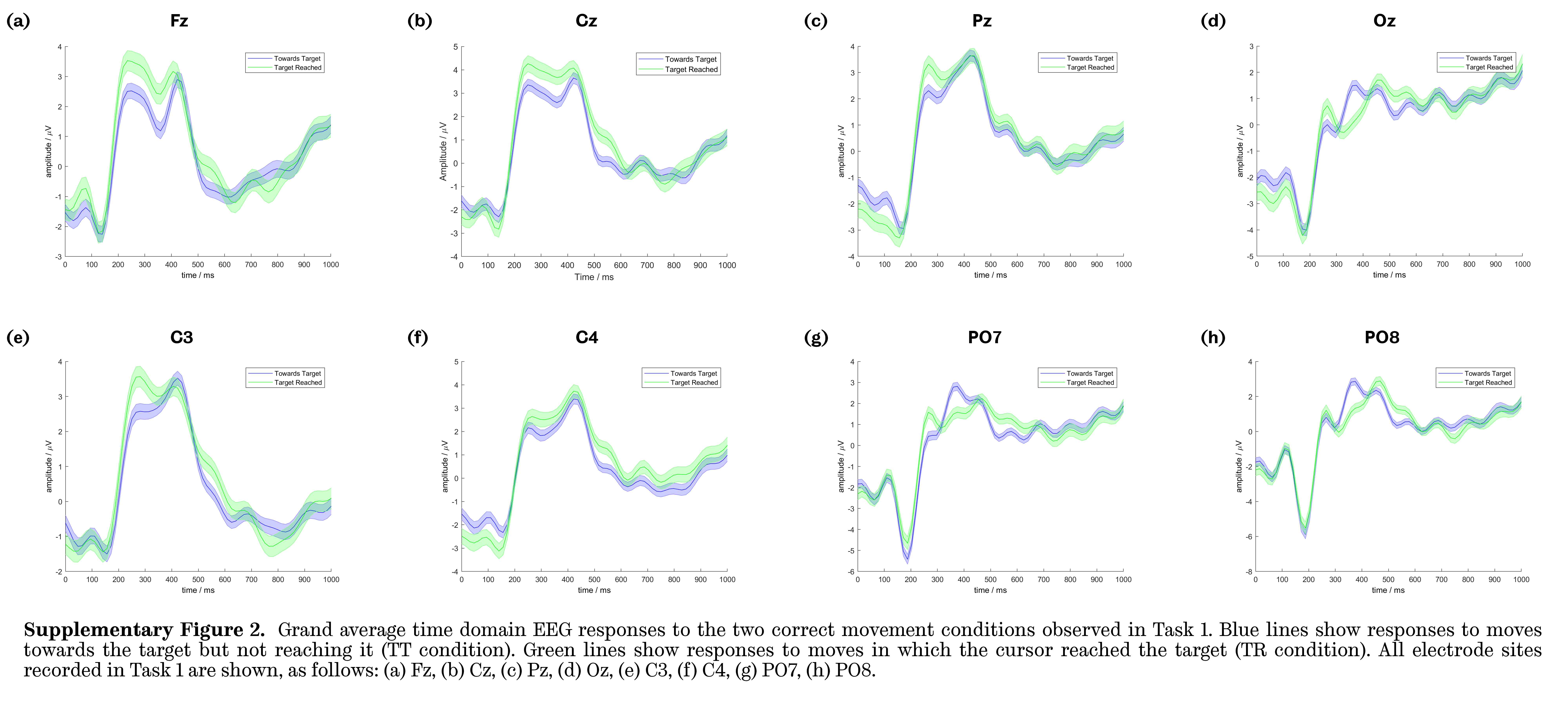

Supplement: Supplementary file 2 [file Image_2.JPEG]
